# Survey on examining prevalence of paternal anxiety and its risk factors in perinatal period in Hong Kong: a longitudinal study

**DOI:** 10.1186/s12889-015-2436-4

**Published:** 2015-11-16

**Authors:** Y.W. Koh, A.M. Lee, C.Y. Chan, D.Y. T. Fong, C.P. Lee, K.Y. Leung, C.S. K. Tang

**Affiliations:** Department of Psychiatry, LKS Faculty of Medicine, The University of Hong Kong, Pokfulam, Hong Kong; School of Nursing, The University of Hong Kong, Pokfulam, Hong Kong; Department of Obstetrics & Gynaecology, The University of Hong Kong, Pokfulam, Hong Kong; National University of Singapore, Singapore, Singapore

**Keywords:** Paternal mental health, Anxiety, Risk factors

## Abstract

**Background:**

There is emerging evidence of the significance of paternal mental health problems among the expectant fathers during the antenatal and postnatal period. The present study aims at determining the prevalence of paternal perinatal anxiety and identifying its risk factors among the fathers.

**Methods:**

A total of 622 expectant fathers were recruited in Hong Kong. The expectant fathers were assessed using standardized and validated psychological instruments on three time points including early pregnancy, late pregnancy and 6 week postnatal. Independent samples *t*-test, one way ANOVA, Pearson’s correlation and multiple linear regression were used to examine the effect of hypothesized risk factors. Hierarchical multiple regression and mixed effect model were also conducted with potential confounding factors controlled for.

**Results:**

Results showed that a significant proportion of expectant fathers experienced anxiety during the perinatal period. Low self-esteem and poor social support were found to be risk factors of paternal anxiety across pregnancy to postnatal period. Work-family conflict could significantly predict paternal anxiety in the pregnancy period.

**Conclusions:**

The present study points to the need for greater research and clinical attention to paternal anxiety, given that it is a highly prevalent problem and could be detrimental to their partner’s well-being and children development. The present findings contributes to the theoretical understanding of the prevalence and risk factors of paternal perinatal anxiety and have implications for the design of effective identification, prevention, and interventions of these clinical problems.

## Background

The perinatal period is a potentially stressful period in which can precipitate the development of mental health problems, including depression and anxiety among vulnerable parents. Compared to maternal mental health problems which had been extensively studied, paternal mental health is an area that is largely under-researched. Nevertheless, various preliminary studies showed that mental health problems are prevalent among expectant fathers [1–4]. There is also emerging evidence of the significance of mental health problems among the fathers in the postnatal period. A study which used the Edinburgh Postnatal Depression Scale (EPDS) to measure paternal depression at 6 weeks postnatal showed a prevalence rate of 9.0 and 5.4 % at 6 months postnatal [2], Another study on 205 expectant fathers that followed up the fathers for 1 year postnatal showed a 11 % of prevalence rate using EPDS [3]. Moreover, a meta-analysis of 43 studies showed that antenatal and postnatal depression was evident in about 10 % of men [5] and was significantly associated with maternal depression [6]. However, the investigation of paternal anxiety is an area that is under-studied compared to paternal depression. Matthey et al. [7] included the diagnostic assessment for panic disorder and acute adjustment disorder with anxiety in a study conducted on expectant parents. They found that the rates of caseness increased by three folds in expectant mothers and three to six folds in expectant fathers over the rates for major or minor depression [7]. This study highlighted the need to assess anxiety in expectant fathers as it is widely recognized that paternal mental ill health could increase the risk of behavioral and emotional problems in children regardless of mother’s mood state [8, 9].

Preliminary studies showed that some risk factors associated with paternal antenatal psychological distress included poor marital relationship, poor social network and insufficient information about pregnancy and childbirth [10]. Having an unsupportive relationship, marital disharmony, being unemployed, young age, poorer social functioning and past history of psychiatric disorder were also found to be associated with paternal mental health problems during the perinatal period [2, 11–13]. Matthey et al. [14] argued that adjustment to parenthood was related to different variables at different times of the perinatal period. It is therefore important to examine risk factors using a longitudinal design. However, to the best of our knowledge, there is no longitudinal study in the existing literature specifically on risk factors identification with respect to paternal anxiety. By identifying risk factors and their relationship with paternal anxiety in a longitudinal manner, the present study would be able to render findings that would impact on our conceptual understanding of the difficulties faced by expectant fathers as well as aid in the development of clinical preventive strategies to improve paternal well-being.

There is, in general a significant research gap on the prevalence and risk factors of paternal anxiety which the present study aims to fill.

## Methods

A prospective longitudinal design using the survey approach was used to systematically investigate the prevalence and risk factors of paternal anxiety across three significant perinatal stages. A total of 622 expectant couples were recruited and followed up from March 2010 till September 2012.

### Subjects

A consecutive sample of 622 expectant fathers and mothers were recruited from two regional hospitals in Hong Kong. The inclusion criteria included: [1] expectant fathers and mothers [2] of Chinese ethnicity [3] able to read and write Chinese (including Mandarin and Cantonese). The exclusion criteria included: [1] couples who were considering termination of pregnancy [2] with primary residence outside of Hong Kong [3] Miscarriage or stillbirth.

### Procedure

The study was approved by the Institutional Review Boards of the University of Hong Kong /Hospital Authority Hong Kong West Cluster. All eligible expectant fathers and mothers attending the antenatal clinic at their partner’s first antenatal presentation were invited to participate in the study. A range of gestational weeks was permitted due to the big variance on gestational week of the couples’ first presentation in the antenatal clinic. Subjects were informed about the objective, background and procedure of the research and that their responses will be kept strictly confidential from their partners. Upon providing informed written consent, subjects completed a set of self-administered questionnaire. A consecutive sample of 622 expectant fathers and mothers were administered a set of questionnaires at their partner’s first presentation (12 weeks gestation of pregnancy) in the antenatal clinic. The questionnaire was completed by the expectant fathers and mothers separately during the waiting time in the antenatal clinic. In the current study, expectant mothers were asked to complete the questionnaires on perinatal depression and anxiety. They were re-assessed when the partners’ pregnancy progressed to 36 weeks and again at 6 weeks after childbirth. In all, the participants were assessed on three time-points. The three time-points were chosen because they coincided with typical medical follow-up and typical assessment time-points in the literature, thus facilitating assessment, response rate and comparison with findings in the literature.

## Measurement

### Paternal anxiety

The Anxiety Subscale of Hospital Anxiety and Depression Scale (HADS) [15] was used to assess perinatal anxiety symptoms among the expectant fathers and mothers. The HADS has been validated for use among medical as well as general populations [16, 17]. It is a 14-item self—administered instrument comprising seven anxiety items and seven depression items from which separate anxiety and depression sub-scale scores are calculated. The anxiety subscale was used in the proposed study. Respondents were asked to respond to the seven anxiety items on a four-point scale. The recommended cut-off of 7/8 was used to identify a probable case of anxiety. HADS-Anxiety subscale gave a specificity of 0.78 and a sensitivity of 0.90. The Cronbach’s alpha of HADS ranges from 0.81 to 0.90. The Chinese version was used in the present study, It showed good internal consistency with a Cronbach’s alpha of 0.77 for the anxiety subscale [17, 18].

## Measurement of risk factors

### Demographic variables

Demographic risk factors including age, educational level, marital status and family income were assessed at first assessment using self-constructed scale. Psychosocial risk factors including social support, self-esteem, relationship satisfaction and work family conflict were assessed at all three time points. Unplanned pregnancy as a psychosocial risk factor was assessed at the first trimester.

## Psychosocial risk factors

### Planned/unplanned pregnancy

Expectant fathers were asked whether the pregnancy was planned or unplanned.

### Social support

Social support was measured with the Multidimensional Scale of Perceived Social Support which consisted of 12 items measuring perceived social support from family, friends, and significant others [19]. The Cronbach’s alpha of the significant other, family and friends subscales were 0.91, 0.87, and 0.85 respectively. The reliability of the scale as a whole was 0.88 which demonstrated a good internal consistency of the scale. The test-retest reliability for the significant other, family and friends subscales were 0.72, 0.85, and 0.75, respectively and 0.85 for the whole scale. The validated Chinese version was used in the present study [20]. The Cronbach’s coefficient alphas of the MSPSS-C scale were 0.89 as a whole, and 0.94 and 0.86 for the friend and family subscales respectively.

### Self esteem

The 10-item Rosenberg Self Esteem Scale (RSE) [21] was used to assess global self-esteem. It consists of ten statements related to overall feelings of self-worth or self-acceptance. The items are answered on a four-point scale ranging from strongly agree to strongly disagree. The RSE has demonstrated good reliability and validity across a large number of different sample groups and has been validated for use with both male and female adolescent, adult and elderly populations. The Cronbach’s alpha of the RSE is 0.92.

### Work-family conflict

The Work- Family Conflict Scale [22] is a 10-item scale developed to measure participants’ perception of work-to-family and family-to-work interference. Sample items are ‘The demands of my work interfere with my home and family life”, and “The demands of my family or partner/partner interfere with work-related activities”. Respondents was asked to respond on a 7-point scale, ranging from 1 “strongly disagree” to 7 “strongly agree”. High scores on the subscales represent high levels of respective direction of work family interference. The Cronbach’s alpha of the Work-Family Conflict scale ranged from 0.82 to 0.90. The scale was used to assess perceived conflict between work and family.

### Relationship satisfaction

The validated 3-item Kansas Marital Satisfaction Scale [23, 24] was used to measure relationship satisfaction. The Cronbach’s alphas are 0.98 and 0.92 for the English and Chinese versions of Kansas Marital Satisfaction Scale respectively.

### Maternal-related risk factors

Partner’s depression and anxiety as measured by the Edinburgh Postnatal Depression Scale (EPDS) and the anxiety subscale of the (Hospital Anxiety and Depression Scale) HADS respectively by the expectant mothers. The Edinburgh Postnatal Depression Scale (EPDS) [25] was used to assess maternal depressive symptoms in the antenatal and postnatal periods. It is a self-report measure consisting of ten items and each item is rated on a four-point scale. It is a well-validated and the most widely used screening measure of postnatal depression among women. It has also been validated for use in the antenatal period [26] and among men as measure of paternal depression [4]. The Chinese version of EPDS has been validated among pregnant women with satisfactory psychometric properties [27]. The recommended cut-off of 12 was used to define a probable case of depression. Cronbach’s alpha for the EPDS is 0.87.

All the measurement tools were summarized in Table 1.

**Table 1:** Summary of the scales

Paternal Anxiety	The Hospital Anxiety and Depression Scale (HADS)—Anxiety Subscale
Demographic Risk Factors	
Age, Educational Level, Marital Status, Family Income	Self-Constructed Scales
Psychosocial Risk Factors	
Unplanned Pregnancy	Self-Constructed Scale
Social Support	The Multidimensional Scale of Perceived Social Support
Self Esteem	The 10-item Rosenberg Self Esteem Scale
Work Family Conflict	The Work- Family Conflict Scale
Marital Satisfaction	The 3-item Kansas Marital Satisfaction Scale
Maternal-related Risk Factors	
Partner’s Depression	The Edinburgh Postnatal Depression Scale (EPDS)
Partner’s Anxiety	The Hospital Anxiety and Depression Scale (HADS)—Anxiety Subscale

### Statistical analysis

The statistical package for the social sciences (SPSS) was used for all analyses. The overall level of significance was taken as 5 % and all estimates were accompanied by 95 % confidence intervals. Descriptive statistics was presented by means and SDs for continuous variables and percentages for categorical variables. Independent samples *t*-test, one way ANOVA, Pearson’s correlation and multiple linear regression were used to examine the effect of hypothesized risk factors. Hierarchical multiple regression were also conducted with potential confounding factors controlled for. Mixed effect model was used to examine the relationships between the mental health problems and different time points of perinatal period.

## Results

### Response rate

A total of 622 expectant fathers and mothers were recruited in the present study at first presentation to the maternity clinic. The response rate was 72.6 %. At 36 weeks of gestation, 337 (54.2 %) completed the questionnaires for both time-points, yielding an attrition rate of 45.8 %. At 6 weeks postnatal, 150 (24.1 %) participants dropped out from the study. A total of 187 (30.1 %) participants completed all three time points of the survey.

### Attrition analyses

An attrition analysis was conducted between the group of participants who completed both antenatal time points (*n* = 337) and the group of participants who dropped out at 36 gestation weeks (*n* = 285).

Bonferroni correction was used to address the problem of multiple comparisons. The significance level of 0.05 was adopted in the current study. Using Bonferroni correction, each category of variables (demographic, psychosocial risk factors and main outcome variables) was tested at a significance level of 0.05/n (n being the number of variables which were examined in each category).

After Bonferroni correction, results showed that there was no significant difference in both the risk factors and main outcomes between those who completed both antenatal time points and those who dropped out at 36 weeks gestation (Table 2).

**Table 2:** Sample characteristics and attrition analysis of expectant fathers with comparison between fathers who completed both antenatal time points and fathers who dropped out in late pregnancy

	Participants who dropped out at 36 weeks gestation (*n* = 285)		Participant who completed both antenatal time points (*n* = 337)		
					Significance level with Bonferroni correction :0.05/5 = 0.01
*Demographic Risk Factors*					
	Mean	SD	Mean	SD	
Age	(18)				
	33.86	5.04	34.47	5.34	*t* (602) = −1.45, ***p = 0.15***
	N	%	N	%	
Marital Status	(3)				
Married/Cohabitating	281	45.18	331	53.22	χ^2^ (1, *N* = 619) = .026, ***p = 0.87***
Divorce/Single	3	0.48	4	0.64	
Parity	(15)				
Primigravida	191	30.71	220	35.37	χ^2^ (1, *N* = 607) = 0.52, ***p = 0.47***
Multigravida	85	13.67	111	17.85	
Education level	(2)				
Secondary or below	128	20.58	132	21.22	χ^2^ (1, *N* = 620) = 2.12, ***p = 0.15***
Tertiary or above	156	25.08	204	32.80	
Family Income	(20)				
<20,000	46	7.40	57	9.16	χ^2^ (2, *N* = 602) = 0.58, ***p = 0.75***
20,000-30,000	73	11.74	77	12.38	
>30,000	158	25.40	191	30.71	
					Significance level at Bonferroni correction :0.05/5 = 0.01
*Psychosocial risk factors*					
Planned/unplanned pregnancy	(7)				
Planned pregnancy	219	35.21	276	44.37	χ^2^ (1, *N* = 615) = 1.69, ***p = 0.19***
Unplanned pregnancy	61	9.81	59	9.49	
Marital dissatisfaction	(12)				
Distressed with Marital	42	6.75	45	7.23	χ^2^ (1, *N* = 610) = 0.34, ***p = 0.56***
Satisfied with Marital	235	37.78	288	46.30	
	Mean	SD	Mean	SD	
Self Esteem	(24)				
	20.71	4.60	21.43	4.50	t (598) = −1.95, ***p = 0.05***
Social Support	(14)				
	61.77	14.77	63.48	13.65	t (608) = −1.49, ***p = 0.14***
Work family conflict	(39)				
	30.63	11.90	30.36	11.70	t (583) = −1.95, ***p = 0.05***
*Maternal-related risk factor*					*Significance level at Bonferroni correction :0.05/2 = 0.025*
	Mean	SD	Mean	SD	
Partners’ depression	(68)				
	8.04	4.69	7.20	4.14	t (554) = 2.23, ***p = 0.026***
Partners’ anxiety	(71)				
	5.02	3.51	4.46	3.38	t (551) = 1.90, ***p = 0.058***
*Baseline Main outcome variables*					
Mental health problems	Mean	SD	Mean	SD	
Anxiety	(18)				
	3.56	3.59	3.52	3.50	t (604) = .13, ***p = 0.90***

Attrition analysis was also conducted between the group of participants who completed all time points (*n* = 187) and the group of participants who dropped out at 6 weeks postnatal (*n* = 150).

Again, results showed that there is no significant difference in risk factors and main outcomes between those who completed all three time points and those who dropped out at 6 weeks postnatal (Table 3).

**Table 3:** Sample characteristics and attrition analysis of expectant fathers with comparison between fathers who completed all time points and fathers who dropped out at 6 week postpartum

	Participants that dropped out at 6 weeks postpartum (*n* = 150)		Participant that completed all 3 time points (*n* = 187)		
					Significance level with Bonferroni correction :0.05/5 = 0.01
*Demographic Risk Factors*					
	Mean	SD	Mean	SD	
Age	(13)				
	33.95	4.94	34.88	5.61	*t (322)* = −1.56, ***p = 0.12***
	N	%	N	%	
Marital Status	(2)				
Married/Cohabitating	146	43.32	185	53.22	χ^2^ (1, *N* = 335) = 1.53, ***p = 0.22***
Divorce/Single	3	0.89	1	0.30	
Parity	(6)				
Primigravida	101	29.97	119	35.31	χ^2^ (1, *N* = 331) = .38, ***p = 0.54***
Multigravida	47	13.95	64	18.99	
Education level	(1)				
Secondary or below	59	17.51	73	21.66	χ^2^ (1, *N* = 331) = .011, ***p = 0.92***
Tertiary or above	90	26.71	114	33.38	
Family Income	(12)				
<20,000	21	6.23	36	10.68	χ^2^ (2, *N =* 325) = 3.67, ***p = 0.16***
20,000-30,000	30	8.90	47	13.95	
>30,000	93	27.60	98	29.08	
					Significance level with Bonferroni correction :0.05/9 = 0.0056
*Psychosocial risk factors*					
	N	%	N	%	
Planned/unplanned pregnancy	(2)				
Planned pregnancy	115	34.12	161	47.77	χ^2^ (1, *N* = 335) = 5.01, ***p = 0.025***
Unplanned pregnancy	34	10.10	25	7.42	
Baseline Marital dissatisfaction	(4)				
Distressed with Marital	17	5.04	28	8.31	χ^2^ (1, *N* = 333) = 0.86, ***p = 0.36***
Satisfied with Marital	130	38.58	158	46.88	
Marital dissatisfaction in late pregnancy	(13)				
Distressed with Marital	18	5.34	29	8.61	χ^2^ (1, *N* = 324) = .84, ***p = 0.36***
Satisfied with Marital	126	37.39	151	44.81	
	Mean	SD	Mean	SD	
Baseline Self Esteem	(14)				
	21.49	4.68	21.39	4.36	t (322) = −1.56, ***p = 0.12***
Self Esteem in late pregnancy	(8)				
	64.82	4.53	21.44	4.36	t (327) = 1.59, ***p = 0.64***
Baseline Social Support	(8)				
	64.82	13.08	62.42	14.03	t (327) = 1.59, ***p = 0.11***
Social Support in late pregnancy	(5)				
	63.76	13.45	61.36	14.78	t (330) = 1.52, ***p = 0.13***
Baseline Work family conflict	(21)				
	30.52	12.02	30.24	11.47	t (314) = .21, ***p = 0.83***
Work family conflict in late pregnancy	(25)				
	31.22	12.04	30.88	12.96	t (310) = .24, ***p = 0.81***
Maternal-related risk factor					Significance level at Bonferroni correction : 0.05/4 = 0.013
	Mean	SD	Mean	SD	
Baseline Partners’ depression	(29)				
	6.90	4.20	7.45	4.10	t (306) = −1.16, ***p = 0.25***
Partners’ depression in late pregnancy	(31)				
	6.92	4.46	6.77	4.05	t (304) = .31, ***p = 0.76***
Baseline Partners’ anxiety	(29)				
	4.19	3.31	4.67	3.43	t (306) = −1.25, ***p = 0.21***
Partners’ anxiety in late pregnancy	(22)				
	4.71	3.46	4.51	3.22	t (313) = .54, ***p = 0.59***
					Significance level with Bonferroni correction :0.05/2 = 0.0025
*Main outcome variables*					
Mental health problems	Mean	SD	Mean	SD	
Baseline Anxiety	(6)				
	3.29	3.28	3.70	3.66	t (329) = −1.07, ***p = 0.29***
Anxiety in late pregnancy	(16)				
	3.84	3.47	3.78	3.08	t (319) = .18, ***p = 0.86***

### Sample characteristics

All of the expectant fathers were recruited from 12 to 28 weeks gestation of pregnancy and the average gestational week was 15.7 week. The mean age of the expectant fathers was 34.19, and the age range was relatively large, ranging from 19 to 55 years old. Among them, 19 (3.1 %) were below the age of 25 years old, 310 (51.3 %) were between 26 to 34 years old and 275 (45.5 %) of them were above 35 years old.

Concerning the fathers’ marital condition, most of the expectant fathers (612, 98.9 %) were either married or cohabitating with their partners, and a small proportion of them (1.1 %) of them reported being single or divorced.

A total of 411 (67.7 %) of the expectant fathers were first-time fathers, and 196 (32.3 %) were experienced father, having at least one child before. Most of them (99.3 %) reported no history of psychiatric illness, while 0.7 % of them had a history of psychiatric illness, among them, majority suffered from depression.

Most (58.1 %) of the expectant fathers were considered highly educated, having received tertiary or education or above. Less than half (41.0 %) of the expectant fathers finished secondary school education only and only. Ninety percent of them finished low primary school education. Due to the small number of the sample attaining only primary education, the analyses were done by combining fathers of primary and secondary education level into one group, making the percentage to 41.9 %.

With regard to total family income, 58.0 % of the families received a total monthly income of HKD 30000 (USD 3800) or above, while 24.9 % of them had a total monthly family income ranging from HKD 20000–30000 (USD 2500–3800). A small proportion of the families (17.1 %) reported a relatively low family income of HKD 20000 (USD 2500) or below per month. The summary of the sample characteristics can be viewed in Table 4.

**Table 4:** Sample characteristics of expectant fathers of in the present study

Characteristics		
	n	%
Age	(18)	
< 25	19	3.1
26–34	310	51.3
> 35	275	45.5
Mean age = 34.19, SD = 5.21		
Marital Status	(3)	
Married/Cohabitating	612	98.9
Single/Divorced	7	1.1
History of psychiatric illness	(9)	
Yes	4	0.7
No	609	99.3
Parity	(15)	
Primigravida	411	67.7
Multigravida	196	32.3
Education Level	(2)	
Secondary	260	41.9
Tertiary or above	360	58.1
Family income	(20)	
< 20,000	103	17.1
20,000–30,000	150	24.9
> 30,000	349	58.0

#### Prevalence rate of anxiety of expectant fathers in early, late pregnancy and 6 weeks postnatal

Expectant fathers’ anxiety was assessed with the anxiety subscale of the Hospital Anxiety and Depression Scale (HADS) [15]. The recommended cut-off 7/8 was used to screen for probable cases of anxiety and 12/13 was used to screen for significant cases of anxiety [15].

**Fig. 1:**
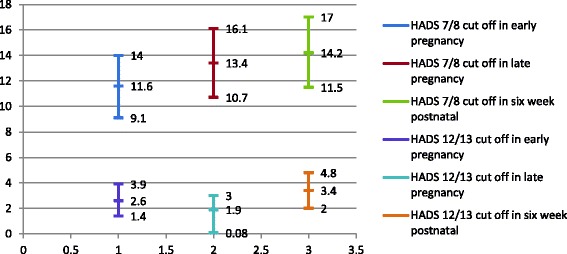
Prevalence of paternal anxiety using hads anxiety cut off 7/8 and cut off 12/13 from pregnancy to 6 week postnatal (including 95 % confidence interval)

Based on the cut- off of 7/8, 11.6 % (95 % CI = 9.1–14.0) of the fathers were found to be probable cases of anxiety in the early pregnancy, the prevalence rate elevated to 13.4 % (95 % CI = 10.7–16.1) in the late pregnancy, and continued to elevate to 14.2 % (95 % CI = 11.5–17.0) at 6 weeks postnatal. The pattern was different for 12/13 cut-off with late pregnancy having the highest prevalence rate at 2.6 % (95 % CI = 1.4–3.9) for early pregnancy, 1.9 % (95 % CI = 0.08–3.0) for late pregnancy, and 3.4 % (95 % CI = 2.0–4.8) for 6 weeks postnatal (Fig. 1).

#### Identification of risk factors for paternal anxiety in early pregnancy, late pregnancy and 6-week postnatal

Demographic, psychosocial and maternal-related risk factors with paternal anxiety across different time points were tested.

Demographic risk factors were first examined. The significant demographic risk factors were treated as confounders while the psychosocial risk factors were tested in the second step. The maternal-related risk factors were also tested with the effect of demographic confounders controlled for. Finally, all the potential risk factors were included into the model by using the multivariate analyses. The results are summarized in Tables 5 and 6.

**Table 5:** Univariate analyses of demographic, psychosocial and maternal-related risk factors for paternal anxiety in early, late pregnancy and 6 week postpartum

	Early Pregnancy				Late Pregnancy			6 Week Posnatal	
Hospital Anxiety and Depression Scale Anxiety subscale (HADS-A)									
Demographic risk factors									
	Mean (SD)	*t*	*p*	Mean (SD)	*t*	*p*	Mean (SD)	*t*	*p*
Parity		1.30	0.20		0.84	0.40		0.41	0.68
Primigravida	3.67 (3.64)			3.89 (3.37)			3.92 (3.73)		
Multigravida	3.27 (3.19)			3.57 (3.04)			3.70 (3.39)		
Education Level		−0.21	0.83		−0.70	0.48		−0.22	0.83
Secondary	3.50 (3.75)			3.66 (3.16)			3.76 (3.85)		
Tertiary or above	3.56 (3.32)			3.92 (3.32)			3.87 (3.45)		
		*F*	*p*		*F*	*p*		*F*	*p*
Family income (HK$/month)		0.18	0.84		1.33	0.27		2.14	0.12
<20,000	3.58 (3.88)			3.17 (2.77)			2.85 (3.41)		
20,000–30,000	3.69 (3.80)			3.96 (3.35)			4.36 (3.82)		
>30,000	3.49 (3.24)			3.97 (3.36)			3.98 (3.52)		
	*N*	*r*	*p*	N	*r*	*p*	N	*r*	*p*
Age	588	−0.06	0.15	308	−0.11	0.06	198	−.002	0.98
Psychosocial risk factors									
	Mean (SD)	*t*	*p*	Mean (SD)	*t*	*p*	Mean (SD)	*t*	*p*
Planned/unplanned pregnancy		−1.44	0.15		2.26	0.13		.003	0.96
Planned pregnancy	3.45 (3.45)			3.69 (3.13)			3.89 (3.64)		
Unplanned pregnancy	4.00 (3.71)			4.43 (3.77)			3.67 (3.43)		
Marital dissatisfaction		5.33	.000**		14.7	.000**		16.8	.000**
					3			7	
Marital distress	6.04 (4.84)			5.49 (3.58)			6.39 (4.11)		
Marital non distress	3.14 (3.05)			3.51 (3.13)			3.48 (3.36)		
	N	*r*	*p*	N	*r*	*p*	β	*t*	*p*
Self-Esteem	588	−0.33	.000**	314	−0.43	.000**	−0.55	−9.12	.000**
Social Support	597	−0.16	.000**	332	−0.36	.000**	−0.43	−6.66	.000**
Work family conflict	572	0.33	.000**	306	0.45	.000**	0.44	6.54	.000**
Partners’ mental health	N	*r*	*p*	N	*r*	*p*	β	*t*	*p*
Depression	542	0.13	.002*	290	0.13	.025*	0.24	3.21	.002*
Anxiety	539	0.12	.004*	299	0.14	.015*	0.29	4.04	.000**

**Table 6:** Hierarchical multiple regression analysis of risk factors for paternal anxiety in early pregnancy, late pregnancy and 6 week postpartum

				Early Pregnancy		
Hospital Anxiety and Depression Scale Anxiety subscale (HADS-A)						
Model	Adjusted R^2^	*F-value*	β	Adjusted-β	*t*	*P*
1	0.005	1.58				0.18
Age			−0.04	−0.06	−1.31	0.19
Parity			−0.19	−0.03	−0.57	0.57
Educational Level			0.17	0.02	0.50	0.62
Family income			−0.18	−0.04	−0.79	0.43
2	0.22	13.93				0.000**
Unplanned			0.37	0.04	0.89	0.37
Pregnancy						
Marital dissatisfaction			1.23	0.12	2.60	0.01*
Self-esteem			−0.16	−0.21	−4.39	0.000**
Social support			−0.02	−0.10	−2.12	0.03*
Work family conflict			0.06	0.22	4.72	0.000**
3	0.24	12.15				0.000**
Partners’ depression			−0.01	−0.02	−0.22	0.82
Partners’ anxiety			0.13	0.12	1.76	0.10
				Late Pregnancy		
Hospital Anxiety and Depression Scale Anxiety subscale (HADS-A)						
Model	Adjusted R^2^	*F-value*	β	Adjusted-β	*t*	*P*
1	**0.00**	**1.18**				**0.32**
Age			−0.08	−0.13	−2.27	0.24*
Parity			0.21	0.03	0.55	0.59
Education Level			−0.26	−0.04	−0.63	0.53
Family Income			0.47	0.11	1.70	0.09
2	0.33	14.22				0.000**
Unplanned			0.38	0.04	0.78	0.44
Pregnancy						
Marital dissatisfaction			0.31	0.03	0.57	0.57
Self-esteem			−0.20	−0.27	−4.26	0.000**
Social support			−0.05	−0.20	−3.37	0.001**
Work family conflict			0.07	0.27	4.41	0.000**
3	0.34	11.83				0.000**
Partners’ anxiety			0.04	0.05	0.58	0.56
Partners’ depression			0.03	0.03	0.32	0.75
			Six week postpartum			
Hospital Anxiety and Depression Scale Anxiety subscale (HADS-A)						
Model	Adjusted R^2^	*F-value*	β	Adjusted-β	*t*	*P*
1	0.01	0.25				0.91
Age			−0.02	−0.03	−0.45	0.66
Parity			−0.51	−0.07	−0.99	0.33
Education Level			−0.33	−0.05	−0.57	0.57
Family Income			0.28	0.06	0.81	0.42
2	0.38	11.33				0.000**
Unplanned			0.17	0.02	0.25	0.81
Pregnancy						
Marital dissatisfaction			−0.86	−0.08	−1.15	0.25
Self-esteem			−0.24	−0.34	−4.35	0.000**
Social support			−0.06	−0.26	−3.51	0.001**
Work family conflict			0.03	0.13	1.72	0.09*
3	0.40	10.19				0.000**
Partners’ depression			0.03	0.03	0.31	0.75
Partners’ anxiety			0.16	0.16	1.47	0.14

**Significant at 0.001 level (2-tailed)

*Significant at 0.05 level (2-tailed)

### Demographic risk factors

Results showed no significant association between any demographic risk factors and anxiety in early pregnancy and late pregnancy. At 6 weeks postnatal, family income was found to be a significant predictor of anxiety. Although the results in one-way ANOVA was not significant [*F* (2, 196) = 2.14, *p =* 0.12], results of post hoc analyses using LSD criterion showed that there was a significant difference in HADS anxiety scores between the group of fathers with low family income (mean = 2.85, SD = 3.41) and those with moderate family income (mean = 4.36, SD = 3.82)(*p* < .05). The family income was treated as a confounder in the following tests on psychosocial and maternal-related risk factors in 6 week postnatal in the univariate analysis.

### Psychosocial risk factors

All psychosocial risk factors except unplanned pregnancy were found to be strong predictors of antenatal and postnatal anxiety. Independent samples t-tests showed that fathers with marital distress (mean = 6.04, SD = 4.84) had higher level of anxiety compared to fathers without marital distress (mean = 3.14, SD = 3.05) in early pregnancy [t (595) = 5.33, *p* = <.001]. The same pattern followed through in late pregnancy, with marital distressed fathers (mean = 5.49, SD = 3.58) having higher anxiety compared to marital satisfied fathers (mean = 3.51, SD = 3.13) [*F*(1, 307) = 14.73*, p* < .001]. At 6 weeks postnatal, marital distressed fathers (mean = 6.39, SD = 4.11) also tended to be more anxious than the fathers without marital distress (mean = 3.48, SD = 3.36), even after controlling for family income [*F(1, 193) = 16.87, p < .001*].

Pearson correlational analyses showed that lower self-esteem was significantly associated with higher anxiety [*r(588)* = −0.33, *p* < .001]. The same pattern of relationship was observed in late pregnancy [*r(314)* = −0.43, *p* < .001], and at 6 weeks postnatal, with poor self-esteem associated with higher anxiety after controlling for the effect of family income (β = −0.55, *t* = −9.12, *p* < .001).

Negative associations between perceived social support and anxiety were found across the 3 time points. In early pregnancy, the association between social support and anxiety was significant [*r(597)* = −0.16, *p* < .001]. Higher anxiety level was also significantly associated with poorer social support in late pregnancy [*r(332)* = −0.36, *p* < .001]. At 6 weeks postnatal, the association between social support and anxiety was remained significant after controlling for the effect of family income [β = −0.43, *t* = −6.66, *p* < .001].

Another important psychosocial risk factor examined was work-family conflict. In early pregnancy and late pregnancy, higher work-family conflict predicted higher level of anxiety among the expectant fathers [*r(572)* = 0.33, *p* < .001] and [*r(306)* = 0.45, *p* < .001] respectively. After controlling for demographic confounders, the positive association between work family conflict and anxiety was still observed at 6 week postnatal (β = 0.44, *t* = 6.54, *p* < .001).

### Maternal-related risk factors

Partners’ anxiety and depression were found to be strong predictors for expectant fathers’ anxiety across different time points. Significant positive relationships were found between partners’ and expectant fathers’ anxiety in early pregnancy [*r(539)* = 0.12, *p* < .05], late pregnancy [*r(299)* = 0.14, *p* < .05] and at 6 weeks postnatal [β *=* 0.29, *t* = 4.04, *p* < .001, after controlling for family income].

Higher level of depression in the partners also predicted high level of anxiety in the expectant father across different time points, in early pregnancy [*r(542)* = 0.13, *p* < .05], late pregnancy [*r(290)* = 0.13, *p* < .05] and 6 week postnatal [β = 0.24, *t* = 3.21, *p =* < .05, after controlling for family income].

### Multivariate results

Stepwise multiple regression was conducted with the significant predictors across different time points. All the risk factors were included in the model, with demographic risk factors in block 1, psychosocial risk factors in block 2 and maternal-related risk factors in block 3. In early pregnancy, marital dissatisfaction was positively related to higher level of anxiety in the expectant fathers (β = 1.23, Adjusted β = 0.12, *t* = 2.60, *p* <0.05). Poor self-esteem and social support also predicted higher level of anxiety in fathers, (β = −0.16, Adjusted β = −0.21, *t* = −4.39, *p* < .001, and β = −0.02, Adjusted β = −0.10, *t* = −2.12, *p* = <0.05) respectively. In addition, work family conflict was a significant predictor of anxiety level [β = 0.06, Adjusted β = 0.22, *t* = 4.72, *p =* < .001]. The model yielded an adjusted R square of 0.24, which was statistically significant [*F(*11, 446) = 12.15*, p* < .001].

For late pregnancy, young age was found to be a significant predictor (β = −0.08, Adjusted β = −0.13, *t* = −2.27, *p* <0.05). Poor self-esteem (β = −0.20, Adjusted β = −0.27, *t* = −4.26, *p* <0.001), poor social support (β = −0.05, Adjusted β = −0.20, *t* = −3.37, *p* <0.001), and high level of work family conflict (β = 0.07, Adjusted β = 0.27, *t* = 4.41, *p* <0.001) were found to be significant predictors of anxiety in late pregnancy. The model yielded an adjusted R square of 0.34, which was statistically significant [*F*(11, 235) = 11.83*, p* <0.001].

In 6 week postnatal, poor self-esteem (β = −0.24, Adjusted β = −0.34, *t* = −4.35, *p* <0.001)and poor social support (β = −0.06, Adjusted β = −0.26, *t* = −3.51, *p* <0.001) at 6-weeks postnatal were found to be strong predictors for expectant fathers’ anxiety level. The model was significant, with an adjusted *R*^*2*^ = 0.40 [*F* (11, 151) = 10.19*, p* <0.001].

The results of the multivariate analyses are summarized in Table 6.

#### Paternal anxiety across different time points of perinatal period

**Fig. 2:**
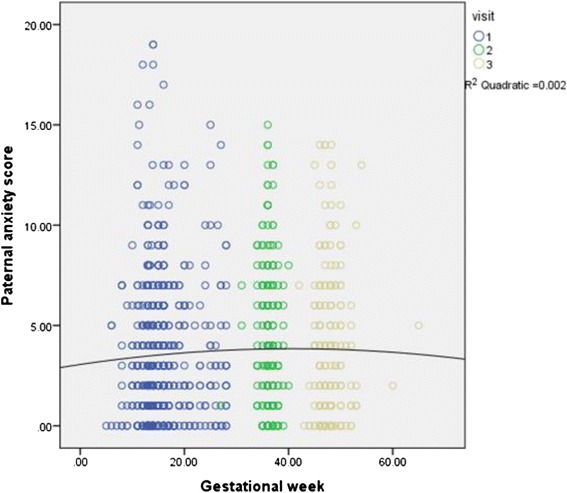
Relationship between gestational weeks and paternal anxiety

Significant positive association was found between the changes of paternal anxiety and gestational weeks (*t* = 2.07, *p* <0.05) after controlling the effects of confounders. The changes of marital dissatisfaction (*t* = 3.62, *p* < .001), self-esteem (*t* = − 7.34, *p* < 0.001), social support (*t* = −3.85, *p* <0.001) and work family conflict (*t* = 5.50, *p* <0.001) were found to be significantly associated with the changes of HADS scores across different measuring time points. The results were shown in Table 7 and Fig. 2.

**Table 7:** Mixed effect model for paternal anxiety across perinatal period (from early pregnancy to six week postpartum)

Parameter	Estimate	Std. Error	df	t	Sig.
Intercept	10.25	1.11	826.86	9.26	0.000
Gestation Week	0.01	0.01	516.34	2.07	0.039*
Marital Status	0.22	1.09	457.88	0.20	0.84
Family Income	−0.04	0.16	461.42	−0.27	0.79
Marital Dissatisfaction	0.16	0.04	844.00	3.62	0.000**
Self Esteem	−0.19	0.03	773.54	−7.34	0.000**
Social support	−0.03	0.01	837.28	−3.85	0.000**
Work Family Conflict	0.05	0.01	836.88	5.50	0.000**
Partners’ Depression (measured by EPDS)	0.04	0.04	794.76	1.02	0.31
Partners’ Anxiety (measured by HADS anxiety subscale)	0.08	0.05	809.82	1.63	0.10

**Significant at 0.001 level (2-tailed)

*Significant at 0.05 level (2-tailed)

## Discussion

According to a large scale survey conducted by the National Institute of Mental Health (NIMH) in 2005, the 12 months prevalence of any anxiety disorder among United States’ male adults was 11 % using DSM-IV criteria [28]. A study that was conducted in Hong Kong in 1993 showed that the prevalence of anxiety symptoms among men were 7.8 % [29] and a review paper in 2006 had shown that the pooled 1-year and lifetime prevalence for total anxiety disorders were 10.6 and 16.6 % [30]. To our best knowledge, there are no studies on the prevalence of anxiety among expectant fathers across the antenatal to the postnatal period using HADS anxiety subscale. In the present study, using the HADS anxiety subscale, the probable cases of paternal anxiety increased as the pregnancy proceed and reached its peak at 6 week postnatal from 11.6 to 14.2 %. One important finding from the present study is that the postnatal period is the high risk period for expectant fathers to develop anxiety and the prevalence of paternal anxiety was not less than maternal anxiety as documented in the literature which is estimated to be 10–15 % [31]. Interestingly, the significant cases of paternal anxiety (using 12/13 cut off) dropped to 1.9 % and went up to 3.4 % in postnatal period. The results showed that fathers reported least prevalence of anxiety in late pregnancy. The fathers who were more anxious about the baby and mothers were expected to be more diligent on the caring of the mother and the baby and gathering the information about delivery during the pregnancy and thus felt more ready for the delivery.

Preliminary studies in the literature showed that the risk factors associated with paternal perinatal psychological distress and mental health problems included poor marital relationship, poor social network and insufficient information about pregnancy and childbirth, past history of psychiatric disorder, young age, and poor social functioning [10, 12, 13]. On the other hand, the demographic and psychosocial risk factors for maternal perinatal anxiety include young age, low socioeconomic levels, low educational levels, primigravida, poor married and family relationships, low self-esteem, negative life events, high perceived stress and poor social support [32–35].

In the present study, univariate analyses found that most of the demographic risk factors were not significant risk factors for paternal anxiety. Interestingly, parity was not found to be a significant risk factor in comparison to the findings on maternal anxiety. Second-time fathers are not that well-equipped psychologically and experience the same level of anxiety (perhaps in different form or different nature) than the first-time fathers. Similar to maternal arm, family income is a predictor of paternal anxiety at 6 week postnatal. One of the most central concepts of men in traditional value system was to be the main provider of the family. Although having children were highly encouraged and thought of as a sign of prosperity in ancient Chinese family, it was too expensive to raise even one child in Hong Kong. The financial burden of raising a child in Hong Kong created greater financial burden on the fathers and thus put them at risk of developing anxiety. In the multivariate results, young age was also found to be significant risk factor at late pregnancy. Fathers who were younger tended to make less money, have less social support and less confidence in being a parent. This result is comparable to the literature regarding maternal mental health problems.

Psychosocial risk factors were found to be strong predictors of higher level of paternal anxiety in present study which is similar to maternal anxiety. The psychosocial risk factors including marital dissatisfaction and poor social support were strongly associated with paternal anxiety from antenatal to postnatal period which were consistent with the findings of previous studies [10, 12, 13]. Cronenwett (1981) argued that men tended to have poorer social support networks compared to women as men tend to rely primarily on their partners for support after getting married [36]. Expectant fathers who had poor social support and relationship dissatisfaction had no one to turn to for advice and support during this transition time which put them at risk of developing anxiety.

Paternal stress is compounded by the fact that resources for parenting are mostly maternal-oriented. As many as 40 % of men stated that they often felt that the information available for them related to pregnancy and childbirth is limited, and that they required more information to prepare them for their role and tasks as fathers. These factors weakened the confidence of the expectant fathers and left them feeling not competent and well-equipped in childrearing.

High level of work family conflict which was not reported in previous literature was found to be a strong predictor of paternal mental health problems across pregnancy. Work family balance was found to be notoriously poor in the families in Hong Kong where the demands of working hours and quality are extremely high. A study on a sample of Hong Kong Chinese parents in dual-earner families indicated that work family conflict was negatively related to job and life satisfaction; the study also showed that the coping behaviors of Hong Kong employed parents were largely ineffective against the problem of work family conflict [37]. The current study filled the gap of knowledge on how work family conflict impacted on expectant fathers’ mental health and hopefully would raise the awareness of the public to pay attention to the importance of maintaining work life balance. Interventions on helping the fathers to cope with poor self-esteem, poor social support, marital dissatisfaction and work family conflict could be very beneficial.

Maternal-related risk factors were also found to significantly predict paternal anxiety at all three time points in univariate analyses. The reciprocal relationship between the mental health of both parties within the couple was documented in previous studies [1, 4]. Maternal anxiety was found to significantly predict paternal anxiety from antenatal to postnatal postnatal period. Maternal depression could also predict high level of anxiety in the fathers in early pregnancy and 6 weeks postnatal.

However, most of the demographic and maternal-related risk factors could no longer predict paternal anxiety in the multivariate model while psychosocial risk factors remained the strongest predictors across different time points except young age was found to be a predictor at late pregnancy This showed that demographic and maternal-related risk factors were weaker predictors compared to psychosocial risk factors. It could be concluded that expectant fathers who had poor self-esteem, poor social support, marital dissatisfaction and high level of work family conflict were more susceptible to anxiety in the perinatal period. These factors undermined the successful transition into the role of father.

In the present study, the prevalence of paternal anxiety across antenatal to postnatal time points was investigated. Changes of scores across time were found in paternal anxiety after controlling for confounders.

Paternal anxiety showed a slight “n” shape for the scores from antenatal to postpartum period. The changes of both scores were significant across three time points. This result indicated that in general, paternal anxiety was more prevalent during pregnancy and the changes over time were significant. Assessment of the expectant fathers’ mental health problems starting from early pregnancy is crucial and multiple assessments throughout the perinatal period is necessary as one-time assessment might limit the detection of perinatal paternal anxiety.

### Limitations

Acknowledgement of the limitations of the present study is essential to make appropriate interpretation and generalization of the results.

A potential problem is that fathers who had non-pregnant partners were not included in the present study as the comparison group. The fathers who accompanied their partners to the maternity clinic might be the group who were more anxious about the pregnancy and thus overestimation might occur in the prevalence of anxiety. Alternatively, men who were in less supportive relationship with their partners may be less inclined to attend the antenatal clinic with their partners. Hence, the reported prevalence rates may well be both an over estimation or underestimation of paternal anxiety in the community. In addition to that, there was no studies which used HADS anxiety score to measure prevalence of paternal anxiety which could provide a legitimate comparison for interpreting the results.

Implications drawn from this study relate mainly to expectant fathers attending antenatal and may not generalizable to the general population. However, the strength of the study lies in its attempt to reach out to expectant fathers in the clinical settling which is much more difficult than reaching out to expectant mother who are much more likely to attend antenatal clinics.

It should also be noted that although the Chinese-Cantonese version of HADS anxiety scale has been well-validated among general population in Hong Kong [18], it has never been used on measuring perinatal paternal well-being in Hong Kong. In addition, the present study used self-report symptoms instruments instead of diagnostic tools to define paternal anxiety. As stated in the literature, males tended to hide the emotions they experienced in comparison to females which justified the under estimation of the rate of males’ mental health problems [38–40]. To minimize the bias, continuous scores for the scales were used in the analyses instead of cut-off scores. To tackle the problems, it should be useful to include diagnostic tools in future studies.

Beside this, the information in present study was collected from single source, namely self-reported questionnaire, which could result in bias. In the future, interviews and qualitative research method with the subjects could fill in the gap that was left. Matched controls should be included in future studies in order to provide a reliable comparison group for the purpose of investigating prevalence rate as well as identifying risk factors. Also, the data was collected from the antenatal clinic of two regional hospitals in the Western and Central districts only. The sampling did not include any subjects from the other public or private clinics or hospitals in the other districts in Hong Kong. Therefore, the population of a relatively lower or higher socio-economic status might be excluded from the present study. Caution should be exercised in generalizing the results to fathers of other backgrounds.

## Conclusion

In conclusion, the rate of paternal perinatal anxiety was shown to be high among Hong Kong Chinese men and should not be overlooked especially in late pregnancy and the postnatal. It should be acknowledged that fathers, being one of the parents, also experience a phase of transition and substantial stress during and after their partners’ pregnancy. Thus, prevention, early identification and intervention of paternal perinatal anxiety are needed.

The present study has also investigated the relationship between paternal antenatal and postnatal anxiety as well as identified significant demographic and psychosocial risk factors for paternal perinatal anxiety. Such knowledge contributes to the effective design of screening, prevention, intervention strategies and also help to identify the high risk groups.

### Ethics committee

The study was approved by The Institutional Review Boards of the University of Hong Kong/Hospital Authority Hong Kong West Cluster.

## Abbreviations

HADS: The Hospital Anxiety and Depression Scale
MSPSS-C: The Multidimensional Scale of Perceived Social Support—Chinese version
RSE: The 10-item Rosenberg Self Esteem Scale
EPDS: The Edinburgh Postnatal Depression Scale
SPSS: The Statistical Package for the Social Sciences
NIMH: National Institute of Mental Health
DSM-IV: Diagnostic and Statistical Manual of Mental Disorders, 4th edition
